# Ultrasound-assisted intrathecal injection of nusinersen in a patient with severe vertebral deformity: a case report

**DOI:** 10.1186/s40981-020-00367-y

**Published:** 2020-08-11

**Authors:** Takashi Nagano, Shinichi Sakura, Noritaka Imamachi, Yoji Saito

**Affiliations:** 1 Department of Anesthesiology, Faculty of Medicine, Shimane University, 89-1 Enya-Cho, Izumo City, Shimane 693-8501 Japan; 2 Surgical Operation Center, Shimane University Hospital, 89-1 Enya-Cho, Izumo City, Shimane 693-8501 Japan

**Keywords:** Ultrasound, Intrathecal, Lumbar puncture, Spinal muscular atrophy, Spinal deformity, Nusinersen

## Abstract

**Background:**

Spinal muscular atrophy (SMA) is a mostly autosomal recessive genetic disease characterized by progressive muscle weakness from anterior horn degeneration. Nusinersen has recently been approved as a disease-modifying drug for SMA that needs to be administered intrathecally. Its injection is often associated with extreme difficulty since patients with SMA have severe vertebral deformity and may be with vertebral instrumentation.

**Case description:**

A 21-year-old female with type 2 SMA and spinal deformity underwent a series of intrathecal injections of nusinersen. The intrathecal injections have been safely and successfully done by using computed tomography imaging and ultrasonography-assisted technique.

**Conclusion:**

This the first report in which ultrasound-assisted technique has been used for the injection of nusinersen through a lumbar puncture in patients with severe spinal deformity. Use of preprocedural ultrasound imaging is highly recommended for treatments that repeatedly require intrathecal access.

## Background

Spinal muscular atrophy (SMA) is a mostly autosomal recessive genetic disease characterized by muscle weakness from anterior horn degeneration. SMA is a progressive disease with variable severity levels depending on its type and is classified into 5 groups from its onset and highest motor function. It ranges from type 0, the most severe form with limited life expectancy, to type 4, the mildest form commonly diagnosed in adulthood [1]. Nusinersen, a recently approved drug for treatment of SMA, does not pass the blood-brain barrier [2] and, thus, needs to be repeatedly administered intrathecally to prevent the disease from progressing. Intrathecal (IT) access is commonly established using the landmark technique in normal subjects. However, patients with SMA are affected by severe scoliosis [3–5] and are often with vertebral instrumentation [3, 4]. In those patients, landmarks for intrathecal access can be difficult to identify, and fluoloscopy and real-time computed tomography (CT)-guided puncture have been shown to help access an intrathecal space [6, 7]. Ultrasound-assisted spinal anesthesia or puncture involves preprocedural ultrasound scanning and comes with less exposure to radiation than fluoroscopy or real-time CT-guided puncture. Here we describe a case with severe vertebral deformity who underwent ultrasound-assisted lumbar intrathecal administration of nusinersen repeatedly.

## Case description

**Fig. 1:**
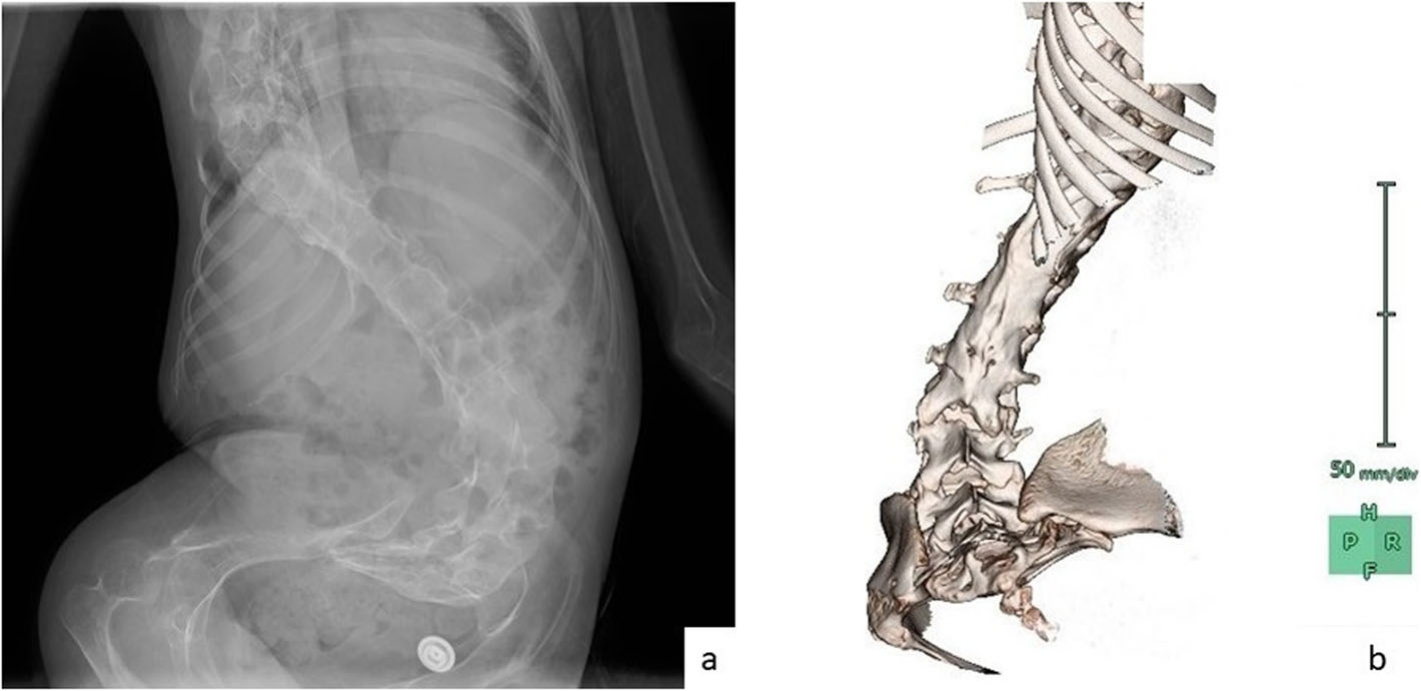
**a** A plain anteroposterior X-ray image of the vertebrae. **b** A three-dimensional reconstructed image of CT scan. A view from the back shows rotation of the vertebrae and fused posterior segment

The patient was a 21-year-old female with type 2 SMA. Neurologists who had been seeing the patient made a plan for her to receive nusinersen, which needs to be intrathecally injected consecutively possibly for the rest of her life. She had a surgical history of growing rod implantation at the age of six which was lengthened 3 times and was removed 4 years later because of infection. She had severe deformity with scoliosis and vertebrae rotation. CT images and plain x-ray images were obtained for preprocedural investigation (Fig. 1). A CT image revealed tight bony fusion in the posterior segment from lower thoracic vertebrae down to L3. The documented Cobb angle was 60° (T5–L5). Because of these complicating factors, she was referred to the anesthesiology department for IT injection of nusinersen. To avoid excessive exposure to radiation, we planned ultrasound-assisted puncture over a CT-guided procedure while taking into consideration the patient’s age.

**Fig. 2:**
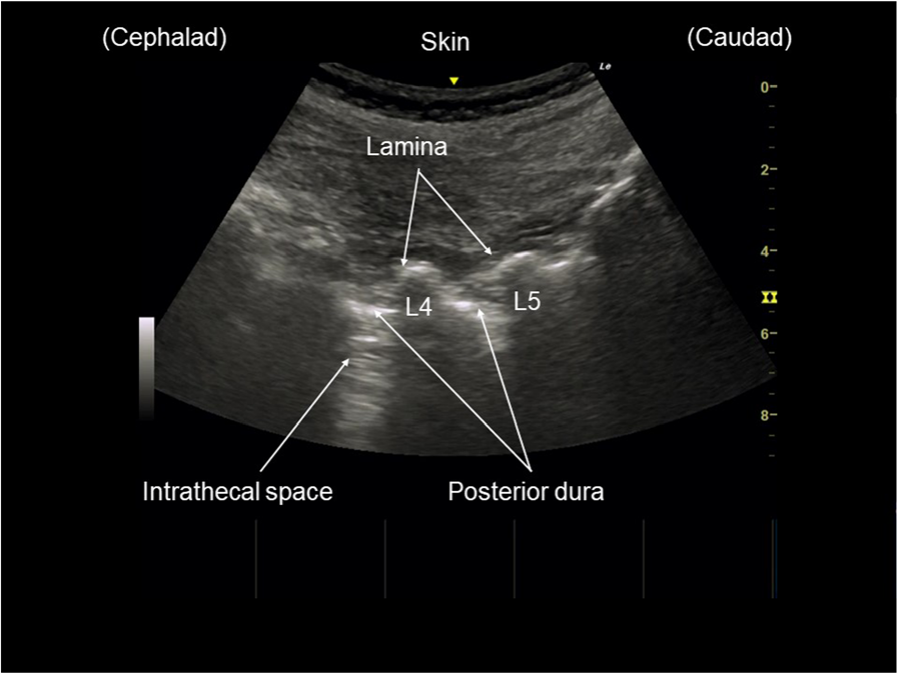
An ultrasound image obtained immediately before puncture when the transducer was placed in a paramedian sagittal oblique plane

**Fig. 3:**
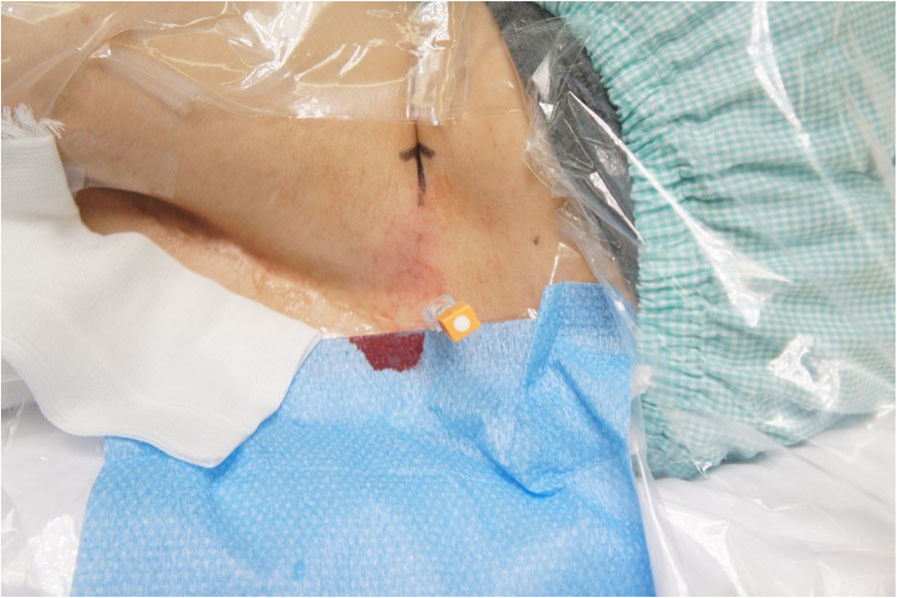
Spinal needle insertion

The procedure took place in a standard operating room. Under sedation with midazolam 1 mg, the patient was placed in the left lateral decubitus position, and the patient was placed in the left lateral decubitus position. Neither the spinous process nor any other part of the spine was palpable in the position. Preprocedural ultrasound scanning was conducted using a 3–8-MHz curved array transducer (LOGIQ e Premium; GE Healthcare, Japan) to find a possible access to the IT space at L3–L4 and L4–L5 (Fig. 2). After local anesthetic infiltration, a 25-G Quincke spinal needle was inserted about 45° to spinous process (paramedian approach) (Fig. 3). On the second attempt after a slight change of course from the first attempt, the needle tip was successfully placed in the IT space with confirmation of a backflow of cerebrospinal fluid. Nusinersen (5 mL, 12 mg) was then injected in approximately 120 s.

No complication was observed during or after the procedure. The patient was later discharged from the hospital. As of May 31, 2020, the patient has successfully received three IT injections in the same way with no trouble (the second and third injections were conducted at 1 and 3 months after the first). The intrathecal administration of nusinersen is planned to continue in half-year intervals throughout her life.

## Discussion

This case report describes a successful establishment of IT access with preprocedural ultrasound imaging (ultrasound-assisted technique). Since spinal anesthesia is usually performed by the landmark technique at a high success rate, ultrasonography is not often employed to perform spinal anesthesia on a daily basis. However, recent reports have shown that using an ultrasound-assisted technique reduces both the number of attempts as well as complication rates [8]. In a systematic review including studies of diagnostic lumber puncture (LP) in emergency departments, ultrasound-assisted LP was associated with a higher success rate, less traumatic LPs, and less procedural time spent [9]. Park et al. reported a reduction of the number of needle passes with ultrasound-assisted spinal anesthesia for patients with mostly mild scoliosis and some moderate to severe scoliosis [10]. Chin et al. also reported a reduction of needle passes with preprocedural ultrasound imaging for patients with obesity, moderate to severe scoliosis, and previous spinal surgery [11].

CT images and X-rays show how the spine looks and where a possible passage to the intrathecal space is. However, these images may not be of help when a patient is positioned for intrathecal puncture. Ultrasound imaging obtained immediately before the puncture with the patient positioned for treatment should improve the understanding of the anatomy and help reduce the number of needle passes and possible complications. This is particularly important when patients might well have to repeatedly undergo spinal punctures.

Intrathecal access is not limited to lumbar spine. In some institutions, cervical punctures have been used for intrathecal nusinersen injections [3, 12]. A recent paper described successful ultrasound-guided cervical punctures for nusinersen administration in adolescents who had severe scoliosis and spinal instrumentation in the lumbar spine [4]. However, cervical punctures are rarely conducted and are challenging with potential risk of spinal cord injury and neurological complications. In contrast, lumbar punctures are much more common and safer and, thus, should be tried first with ultrasound even when the access appears extremely difficult.

We did not conduct a real-time ultrasound-guided procedure due to technical difficulty. In a study that investigated feasibility of real-time ultrasound-guided spinal anesthesia, the authors showed a possible reduction in “difficulty” in patients scheduled for lower limb surgery [13]. Real-time guided puncture has a possible advantage in patients with severe scoliosis because an ultrasound-assisted technique only provides the insertion point and angle that the operator should memorize. The feasibility of using real-time ultrasound-guided technique in patients with severe scoliosis, however, remains to be investigated.

## Conclusion

We describe a series of successful ultrasound-assisted intrathecal injections of nusinersen in a patient with severe vertebral deformity due to SMA. This case report further suggests the functionality of additional use of ultrasound imaging when intrathecal puncture is required in a patient whose intrathecal access is challenging.

## Data Availability

Data sharing is not applicable to this article as no datasets were generated or analyzed during the current study.

## Abbreviations

SMA: Spinal muscular atrophy
IT: Intrathecal
CT: Computed tomography
LP: Lumber puncture
T: Thoracic
L: Lumber
